# Influence analysis and promotion countermeasures of green finance policy in China—Traceability based on grounded theory and validation using the csQCA method

**DOI:** 10.1371/journal.pone.0285862

**Published:** 2023-05-18

**Authors:** Li Junjie, Wen Ke, Zhang Bei, Dai Xin, Qi Meng, Liu Bangfan

**Affiliations:** 1 School of Public Administration, Yanshan University, Qinhuangdao, China; 2 ChinaHebei Public Policy Evaluation and Research Center, Qinhuangdao, China; 3 School of Economics and Management, HanDan College, HanDan; 4 Institute of Marxism, Shandong University, Jinan, China; 5 Institute of Marxism, Chinese Academy of Social Sciences, Beijing, China; Shenzhen University, CHINA

## Abstract

Green finance is closely related to sustainable energy development. Using the NVivo12plus software, a governance model of China’s green finance policy was constructed using 22 green finance policy texts at the central level as research objects. Furthermore, based on the csQCA method, Tosmana software was used to develop and verify a theoretical model of 19 policy text cases. The research results demonstrate that policy belief, policy objectives, policy tools, policy feedback, and policy cycle are the main components of China’s green finance policy governance. Furthermore, policy instruments are the fundamental factors affecting the governance effectiveness of China’s green finance policy. Policy goals and policy feedback dominate the influence pattern of green finance policy in China. There are three modes driving the influence of green finance policies: regulation-oriented, collaborative-driven, and tool-guided. Finally, for the optimization and improvement of green finance policies, three forces must be improved: stimulus force, driving force, and promotion force.

## 1. Introduction

Climate change is a common challenge facing the global population and environment, necessitating the sustainable development of mankind. As such, promoting green development is the inevitable choice for mankind to cope with climate change [1]. In this context, green finance plays a key supporting role, as an important part of green development [2]. In recent years, a series of green finance policies have been introduced in China, with the government increasing its support for green finance policies. Certain achievements have been made regarding green development; however, considering the notable funding gap for China’s "double carbon" development goal, green finance still has significant room for development [3]. Improving green finance policy and the standards system to promote green transformation development not only has great significance for promoting the green transformation, but is also an important issue regarding the development of green finance in our country [4]. Exploring current green financial policy, its internal governance logic, and the interactions and cooperation between its internal components allows for optimization and improvement of the green financial policy system.

Green finance and sustainable development have become an important theme for both academic and practical departments. When people think about sustainable development, it is closely connected with green finance (and vice versa). Therefore, green finance and sustainable development have become inevitably linked. Relevant assertions have been put forward by many scholars, including those stating that green finance models can promote sustainable economic development [5]; developing green finance can help to build ecological civilization and promote sustainable development [6]; the development of green finance can promote sustainable development and contribute to common prosperity [7]; international cooperation on green finance has gradually deepened to promote the sustainable development of the global economy [8]; green finance can promote the sustainable development of energy [9]; green finance promotes green development [10]; practicing green finance can lead to carbon neutrality and sustainable development [11, 12]; improving the green finance system may enhance the sustainable development of commercial banks [13–15]; green finance promotes sustainable investment [16]; the development of green finance must be based on sustainability [17]; developing green finance will support green and low-carbon development [18]; deepening the construction of the green financial market can promote sustainable development [19]; developing green finance will promote industrial structure upgrading and sustainable economic growth [20]; and green finance is the best mode of sustainable development [21]. According to these arguments, green finance can be considered as a kind of sustainable finance. Based on the above conclusions, more and more countries and regions have been paying more attention to green finance and sustainable development, introducing and implementing policies in order to promote the development of green finance in their own countries or regions. In this context, China is no exception. In recent years, China has introduced a series of policies to promote green finance. In the existing literature, previous studies have given more attention to both theoretical and practical analyses of China’s green finance, but there is a lack of research providing specific analyses of China’s green finance policy; that is, at present, studies focused on the analysis of China’s green finance policy texts remain insufficient. The analysis of such policy texts is expected to not only be instructive for the theoretical study of green finance, but also valuable for determining practices which may promote the sustainable development of green finance in other countries or regions. Therefore, this paper uses a mixture of two qualitative research methods—grounded theory and Clear Set Qualitative Comparative Analysis (csQCA), with the help of NVivo and Tosmana software—in order to carry out a systematic analysis of China’s green finance policies, with the aim of contributing to and expanding upon the related literature in an effective manner.

Based on the above considerations, the specific research objectives of this paper are as follows: first, we clarify the basic status quo of the current research and practice of green finance policy in China at the macro level; second, we analyze China’s current green finance policy texts using grounded theory and conduct theoretical modeling of the governance logic, element structure, and internal operation of China’s green finance policy; third, through clear set qualitative comparative analysis (csQCA), the causal correlations are further compared and analyzed, considering the composition of elements and their optimal combination path; and, finally, we obtain optimized promotion paths for green finance policy in China.

With this study, we intend to contribute to the academic literature in the following aspects: on one hand, by systematically sorting out the current policy governance logic of green finance in China, we present policy cases exemplifying China’s green finance development in the field of green finance research; on the other hand, we use two qualitative research methods—namely, grounded theory and Clear set Qualitative Comparative analysis (csQCA)—to conduct hybrid research on China’s green finance policy, thus providing a methodological reference for future research on policy texts.

## 2. Literature review and analysis framework

From the perspective of theory and practice, green finance is a developing field. It is in the process of exploration and development in terms of concept and value, as well as system and mechanism. Even considering the definition of green finance, a consensus has not yet been reached [22]. Green finance can be generally defined as all financial services for green development and sustainable development, focusing on green industry, green economy, and green society [23]. Over the past decade, academic research on green finance at home and abroad has become a prominent scientific field, and there are abundant studies focused on the concept in different academic circles [24]. This not only indicates the demand for green finance theory for green development, but also highlights the enthusiasm and sincerity of the academic community to pay attention to green development. Overall, scientific research in this field presents the following characteristics:

First, five main lines of theory can be observed. The first main line involves the connotation, definition, and value research of green finance [25–27]. The second main line involves the evaluation and measurement of green finance development [28–30]. The third main line involves the study of the impact of green finance development on market entities (e.g., financial institutions, enterprises, and individuals) [31–33]. The fourth main line involves the correlation between green finance and social sustainable development [25, 34, 35]. Finally, the fifth main line is system research, involving the focus, difficulty, and emphasis of the subject [3, 36–38]. Theoretical research on green finance has gradually shifted from connotative to systematic research and from theoretical to practical research [32, 39]. A key line that scholars have more recently focused on is the development, efficiency, and evaluation of green finance [40], as well as how to promote green finance and construct a benign financial ecology [41], in order to construct and improve green finance systems suitable for the development of domestic society and economy.

Second, there is a lot of cross-disciplinary research related to green finance. Scientific research on green finance may integrate such subjects as economics, finance, the environment, chemical industry, law, information, architecture, engineering, culture, and nuclear energy, which require highly practical and skill-based knowledge. Among the most recent representative papers, we found the following: *An outlook on the development of renewable energy*, *policy measures to reshape the current energy mix*, *and how to achieve sustainable economic growth in the post COVID-19 era* (2022) [42]; *The role of green finance in mitigating environmental degradation*: *Empirical evidence and policy implications from complex economies* (2023) [43]; *How do green energy investment*, *economic policy uncertainty*, *and natural resources affect greenhouse gas emissions*? *A Markov-switching equilibrium approach* (2022) [44]; and *How energy transition and environmental innovation ensure environmental sustainability*? *Contextual evidence from Top-10 manufacturing countries* (2023) [45]. At present, most research is focused on the fields of economics, finance, the environment, and law, while less studies are related to the chemical industry, information, architecture, engineering, culture, and nuclear energy fields. Of course, some pioneering research articles in these fields have been published, such as the related research on green finance and nuclear energy including the following two articles: *Does nuclear energy consumption contribute to human development*? *Modeling the effects of public debt and trade globalization in an OECD heterogeneous panel* (2022) [46]; and *Environmental footprint impacts of nuclear energy consumption*: *The role of environmental technology and globalization in ten largest ecological footprint countries* (2022) [47].

Third, there are significant differences between academic studies at home and abroad. Due to differences in political and policy systems, economic development status, social environment, information technology, and other factors, significant differences in the indicators used to measure and evaluate the development level of green finance in different countries and regions can be observed, which makes it difficult for academic circles in different countries and regions to reach a consensus on the scientific concept of green finance [48–50]. Therefore, it is difficult to directly translate and apply green finance research results in different countries and regions. Of course, they may still be used for reference, especially from theoretical, model, and policy governance perspectives. As such, it is well worth gaining a comprehensive view of green finance research results both at home and abroad.

In summary, based on the universality of green development and the necessity of green finance to support green development [51], scholars worldwide have attached great importance to green finance research. Consequently, green finance research results are fruitful and there exists a broad range of research prospects in this field. Given the externality of environmental protection and the profit-driven nature of finance, the importance of policy in the development of green finance is self-evident [52]. On 2022-12-2, a total of 1775 CSSCI and Peking University core papers with the subject "green finance" were retrieved from the database of China National Knowledge Network. Meanwhile, a total of 609 CSSCI and Peking University core papers with the theme of "Green finance with policies" were retrieved. Only 46 core papers from CSSCI and Peking University were retrieved with the title of "Green Finance with Policies." This means that, in terms of the current research status, direct research on green finance policies accounts for a small proportion in the green finance literature. Research focused on the interpretation of green finance policy research based on grounded theory typically draws on a qualitative comparative analysis method. To the best of our knowledge, there exists no research on the interactions, feedback, and synergy within the green finance policy system. Therefore, taking China’s current green finance policy as a research object, examining its governance logic and structure, determining any governance dilemmas, and proposing targeted policy suggestions from the perspective of policy science by taking the Chinese system mechanism and culture in consideration comprises a research contribution which is of great theoretical and practical significance.

**Fig 1:**
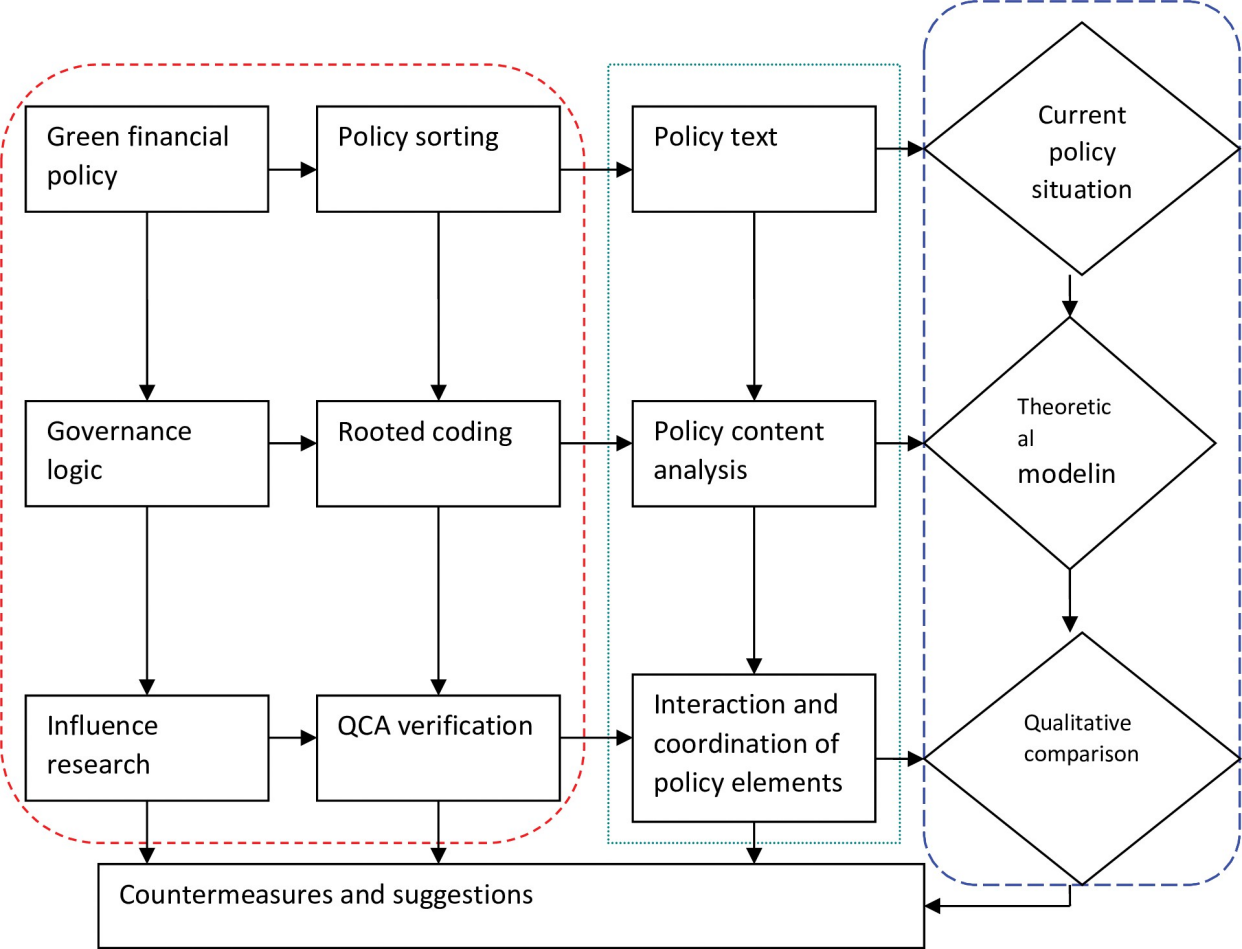
Analysis framework.

Based on the above investigation and analysis, the analysis framework of this paper is presented in the following Fig 1.

## 3. Research methods and data

### 3.1 Research methods

Grounded theory is a scientific research method allowing for theories to be discovered from data. The original intention of grounded theory theoretically orientated and, as such, generating theories from data is the key advantage of grounded theory. In this paper, drawing on grounded theory [53], we use the NVivo12plus software to analyze the content of policy texts [54] allowing for an analysis of the content of China’s current green finance policy texts, consequently allowing us to obtain the main framework, main content, and basic logic underlying the structure, system, and operation of China’s green finance policy. The NVivo 12plus software dutilized in this paper has been successfully used in our previously published articles [55–58].

Qualitative comparative analysis, as a method for comparison of configurations, regards positive social phenomena as different results caused by complex combinations of different attributes. The assessment of multiple concurrent causality is reduced by identifying different causal pathways that lead to the same outcome. The advantages of csQCA are as follows: First, csQCA conforms to the principle of simplicity. Compared with the other two methods for QCA, csQCA is the most widely used in the field of policy research. Based on a Boolean set, csQCA not only can simplify complex social phenomena, but also better preserves potential interests and related phenomena. In addition, in the case of green finance policy, the obtained data are not typical digital data; thus, it is of little significance to set an accurate threshold. Second, csQCA is not limited by the sample size, and the robustness of the obtained results depends on the composition of the sample itself. However, there are only 22 research samples and 5 explanatory variables in this paper, which well meets the suggestion of QCA: "usually select 4–6 explanatory conditions and 10–40 cases" [59]. Third, the method is suitable for explaining multiple complex causalities, rather than linear causalities, which is an advantage well-matched to the characteristics of the considered green financial policy texts. The political, economic, and social aspects of green finance policy design are affected and restricted by compound causality, making it unsuitable for interpretation with regard to linear causality. Fourth, qualitative research is often questioned for its lack of universality and loose structure, while the iterative nature of QCA supports a moderate degree of universality, can realize the systematic comparison of complex cases, can be transformed into logic, and is replicable. Thus, the scientific characteristics of QCA are maintained, reducing the ambiguity and subjectivity inherent to traditional qualitative research methods [60]. Therefore,on the basis of rooted analysis, a QCA method is used in this paper to conduct clear set qualitative comparative analysis based on the action mechanism of the current green finance policy composition logic [61], with the aim of understanding the underlying causality of the interaction between the green finance policy governance elements.

### 3.2 Data collection

For this paper, 22 legal and policy texts related to green finance were retrieved and collected on October 20, 2022, using tools provided by the Chinese government (www.gov.cn), Peking University Talisman, China National Knowledge Network, and so on (see Table 1 for the details of the policy texts).

**Table 1:** List of legal and policy texts.

Serial Number	Symbol Number/Date	Title
N01	CBIRC 15 (2022)	Notice of China Banking and Insurance Regulatory Commission on Issuing Green Finance Guidelines for the Banking and Insurance Industries
N02	27 May 2021	Notice of the People’s Bank of China on the Printing and Distributing of the Green Finance Evaluation Plan for Banking Financial Institutions
N03	PBC 29 (2018)	Notice of the People’s Bank of China on Matters Concerning Strengthening the Supervision and Administration of the Duration of Green Finance Bonds
N04	31 August 2016	The People’s Bank of China, the Ministry of Finance, the Development and Reform Commission and Other Guidelines on Building a Green Finance System
N05	PBC 180 (2022)	Notice of the People’s Bank of China, the Development and Reform Commission, the Ministry of Finance, the Ministry of Ecology and Environment, the Banking and Insurance Regulatory Commission and the Securities Regulatory Commission on Printing and Distributing the Overall Plan for Building a Green Finance Reform and Innovation Pilot Zone in Chongqing
N06	PBC 116 (2019)	Notice of the People’s Bank of China on Supporting the Issuance of Green Debt Financing Instruments in the Green Finance Reform and Innovation Pilot Zone
N07	Bulletin PBC No. 39 (2015)	Announcement of the People’s Bank of China (2015) No. 39—Announcement on Matters Related to the Issuance of Green Finance Bonds in the Interbank Bond Market
N08	NAFMII No. 7 (2022)	The National Association of Financial Market Institutional Investors on Strengthening the Self-discipline Management of Green Finance Bond Duration Information Disclosure
N09	CBRC Office No. 186 (2014)	Notice of the General Office of the China Banking Regulatory Commission on Printing and Distributing the Key Evaluation Indicators for the Implementation of Green Credit
N10	CBRC No. 4 (2012)	Notice of the China Banking Regulatory Commission on Issuing Green Credit Guidelines
N11	CBRC Office No. 40 (2013)	Opinions of the CBRC General Office on Green Credit Work
N12	Announcement of the People’s Bank of China and China Securities Regulatory Commission No. 20 (2017)	Announcement of the People’s Bank of China and the China Securities Regulatory Commission (2017) No. 20—Guidelines on the Evaluation and Certification of Green Bonds (Interim)
N13	Announcement of the China Securities Regulatory Commission No. 6 (2017)	The China Securities Regulatory Commission’s Guidance on Supporting the Development of Green Bonds
N14	PBC 96 (2021)	Notice of the People’s Bank of China, the National Development and Reform Commission and the China Securities Regulatory Commission on Issuing the Catalogue of Projects Supported by Green Bonds (2021 Edition)
N15	Development and Reform Office Finance No. 3504 (2015)	Notice of the General Office of the National Development and Reform Commission on Issuing Guidelines on the Issuance of Green Bonds
N16	China Municipal Cooperative Development No. 172 (2022)	Notice of the China Interbank Market Dealers Association on Matters Related to the Evaluation and Certification Institutions’ Operation of Green Debt Financing Instruments
N17	Bulletin NAFMII No. 10 (2017)	Announcement of the Association of China Interbank Market Traders No. 10 (2017)—Announcement on the Release of the Guidance on Green Debt Financing Instruments for Non-Financial Enterprises and Supporting Forms
N18	JR/T 0227–2021	Guidelines on the Environmental Information Disclosure for Financial Institutions
N19	JR/T 0228–2021	Environmental Rights Financing Vehicle
N20	MEEC Climate 57 (2020)	The Ministry of Ecology and Environment, the National Development and Reform Commission, the People’s Bank of China and other guidelines on Promoting Investment and Financing in Response to Climate Change
N21	26 April 2021	General Offices of the CPC Central Committee and The State Council Issue Opinions on Establishing and Improving the Value Realization Mechanism of Ecological Products
N22	Development and Transformation Energy No. 206 (2022)	Opinions of the National Development and Reform Commission and the National Energy Administration on Improving the Institutions, Mechanisms, Policies and Measures for the Green and Low-Carbon Energy Transition

### 3.3 Sample selection

Saturation has become an important criterion for judging the quality of qualitative research and explaining the rationality of sample sizes [62]. At present, there is no consensus in the academic circle regarding saturation testing in qualitative research, and different scholars have their own standards for the number of theoretical saturation cases, based on their own research experience [63–65]. The latest view was proposed by Hagaman & Wutich in 2017. According to their own research experience, for a relatively homogeneous or specific research object, 16 cases are sufficient to achieve theoretical saturation while, for a study with high heterogeneity, 20–40 cases are required to achieve theoretical saturation [66]. Combined with the actual situation of this study, the text data collected for this study were China’s current green finance policies. Considering the nature of the policy texts, there is a certain homogeneity: from the perspective of the policy texts, green finance policies typically include content related to regulation, guidance, incentive, supply, the environment, and other aspects. After consulting the opinions of experts in the industry, we selected 19 policy texts (within the range of 16–20 samples) as the final sample size to achieve theoretical saturation, while the remaining 3 texts were used as samples for the theoretical saturation test.

## 4. Rooted theory tracing

### 4.1 Root coding analysis

The conducted analysis and processing of policy text was based on the data coding operation process and ideas determined by Glaser et al. [53], including open coding, axial coding, and selective coding. In order to ensure the authenticity and reliability of the case study, and to avoid subjective understanding deviation as much as possible, the text materials used for coding were composed of the collected fragments of the original materials. The specific coding is detailed in the following.

#### 4.1.1 Open coding

Open coding is the process of interpreting disordered raw data in order to discover new insights and generate initial concepts from the phenomena presented by the data. In this paper, we selected 938 original sentences, which were initially conceptualized by labeling them individually. In order to reduce the subjective factors in the research, the original words of the legal and policy texts were used as the codes of the labels as much as possible. The concepts with frequency less than 3 and those which could not be categorized were eliminated, and 30 initial categories were finally formed. These included propaganda leads (9 reference points, same below); practical issues (3); implementing the decisions and arrangements of superiors (18); supporting green, low-carbon, and high-quality development (13); promoting the development of green finance (12); serving economic activities with both environmental and social benefits (7); promoting the transformation and upgrading of the economic structure and the transformation of the economic development mode (7); practicing the concept of green development (5); promoting carbon peak and carbon neutrality (5); addressing climate change (3); product development (393); risk management (56); technological governance (43); international connection (32); pilot demonstration (31); industrial and industrial guidance (26); standard system (25); improving mechanisms (22); capacity building (19); supporting local governments (15); market operation (13); connecting society (10); cooperation promotion (3); policy implementation (3); supervision and management (68); information disclosure (51); incentives and constraints (40); third-party support (26); accountability according to regulations (16); and revision and improvement (4), as shown in Table 2 (due to spatial limitations, only partial results are listed).

**Table 2:** Open coding (partial).

Category (Reference Point/Piece)	Original Representative Statement
Promoting the development of green finance (12)	Promoting the development of the banking and insurance industry (N01)
Tackling climate change (3)	Better leverage of the supporting role of investment and financing in addressing climate change (N20)
Product development (393)	Market investment institutions will be encouraged to develop public and private funds and other green financial products based on the Green Index to meet the needs of investors (N13)
Risk management (56)	Banking and insurance institutions shall urge their clients to strengthen environmental, social, and governance risk management by improving contract terms (N02)
Docking international (32)	Promoting international cooperation in green finance (N04)
Pilot demonstration (31)	Adhere to the first trial; the risk is controllable (N05)

#### 4.1.2 Axial coding

The main task of axial coding is to extract the relationships between categories, according to the coding mode. It is a complex inductive deduction process that connects sub-categories and main categories through multiple steps [67, 68]. Using axial coding, the above 30 categories were further summarized into 17 sub-categories involving policy beliefs and policy objectives, including problem belief, value belief, mission, vision, values, regulatory tools, support service tools, market tools, test tools, innovation tools, disclosure feedback, regulatory feedback, guidance feedback, accountability feedback, third-party feedback, revision cycle, and cohesion cycle. There were five main categories, including standard, policy instrument, policy feedback, and policy cycle, as detailed in Table 3.

**Table 3:** Axial coding.

Category of Principal	Secondary Category	Category of Influence Relationship	Connotation of Relationship
Policy belief	Problem belief Belief in value	A real problem	Policy beliefs include perceptions of important causal relationships and perceptions of the importance of the problem This is the logical starting point of green financial policy governance
		Publicity and guidance	
Policy objectives	Mission Vision Values	Tackling climate change	Beliefs derive goals. Under the guidance of policy beliefs, governments at all levels generate a sense of mission, reach a common vision, decompose the vision into values, integrate values into development strategies, and embody sub-goals. It is the spiritual link of green finance policy governance logic
		Support green, low-carbon, and high-quality development	
		Promote carbon peak and carbon neutral	
		To implement the decisions and arrangements made by superiors	
		Promote green finance	
		To serve economic activities with both environmental and social benefits	
		Promote economic structural transformation and upgrading and change the pattern of economic development	
		Practicing green development	
Policy instrument	Instrument of control Support service tools Tools of market Tools of test Tools for innovation	Standards system	Policy tools are processes by which governments at all levels adopt a series of policy measures and mobilize various resources to organize, coordinate, and control in order to turn vision into reality. They are the key links of green finance policy governance
		Improve the mechanics	
		Policy implementation	
		Risk management	
		International connection	
		Promote cooperation	
		Support places	
		Connected society	
		Capacity building	
		Industry and industry guidance	
		Product development	
		Operation of market	
		Pilot demonstration	
		Technical management	
Policy feedback	Disclosure of feedback	Disclosure of information	Policy feedback is the activity and process of correcting and optimizing policy tools, ensuring the realization of policy objectives, and are an important link of green finance policy governance
	Regulatory feedback	Supervision and administration	
	Guide feedback	Incentives and constraints	
	Feedback on accountability	Accountability according to regulations	
	Third-party feedback	Third-party support	
Policy cycle	Cycle of revision	Revision and improvement	Policy cycle is the process of policy improvement and revision under the influence and promotion of policy feedback results. This is an indispensable link of green finance policy governance. It is not only the last link in this round of policy governance, but is also a new starting point for progressive improvement of governance; that is, the beginning of the next round of policy governance.
	Fit in		

#### 4.1.3 Selective encoding

The purpose of selective coding is to discover a core category from a main category and establish the relationships between the core category and other categories in the manner of a storyline, in order to refine the theoretical research model [69]. This is the final step in the coding analysis and formation of a theoretical framework. The core category determined in this study was “governance logic of China’s green finance policy”, which consisted of five main categories: policy belief, policy objectives, policy tools, policy feedback, and policy cycle.

The storyline surrounding this core category can be roughly understood as follows: the governance logic of China’s green finance policy is a dynamic process driven by policy beliefs. From Sabatier’s division and definition of a belief system, a belief system can be regarded as a set of basic values, a causal hypothesis, and the resulting cognitive system of problems. Policy core beliefs refer to the basic strategy and fundamental policy position concerning deep core beliefs obtained in the policy domain or sub-system. For our country, different levels of government will differ in instrumental belief regarding the secondary aspects of the goal of achieving the core values of the policy; however, the core beliefs regarding the policy remain the same. It is precisely due to the consistency of core policy beliefs that governments at all levels can coordinate their actions, thus leading to the emergence of policy interactions. Put simply, a policy goal is the policy vision with a clear outline reached by governments at all levels under the driving influence of policy belief. It is derivative of policy belief and guides other policy governance links in addition to acting as a policy belief. Policy tools are the specific policy means adopted by governments at all levels to promote the realization of policy objectives. The selection and integration of different types of policy tools is necessary, such as regulation, supply, and consumption. Policy feedback is the process of publicly feeding back the policy results generated by the operation of policy tools to governments at all levels, consequently implementing rewards and punishments. The purpose of performance feedback is to inform the evaluated object (i.e., at all levels of government) whether the pre-determined goals have been achieved, allowing for improvement plans to be made. Policy cycle is an activity and process based on policy feedback, combined with new policy information to help the policy subjects to gradually adjust and improve the used policy tools for the effective achievement of policy objectives.

Specifically, the storyline produced by our country’s green financial policy governance process can be expressed as follows: Against the background of a warming climate and a severe environmental situation, the policy subject realizes the importance of constructing and improving the green finance governance system to address the urgency of giving consideration to both economic development and environmental protection economic activities. Then, through a series of propaganda and educational activities required by certain policies, the policy implementation subject reaches a consensus on developing green finance in our country. Based on this, the concept of green development can be established and practiced, green transformation development can be promoted and, finally, the policy goals of “carbon peak” and “carbon neutrality” can be achieved. Then, a mixture of regulation, support services, market, experiment, innovation, and other policy tools can be used, while various policy feedback forms, such as disclosure, supervision, guidance, and third-party feedback, can be utilized to continuously achieve policy goals and consolidate policy beliefs. At the same time, with the development of green finance practice, the green finance policy system will be constantly revised and improved, and the activities and processes related to connection and coordination with policies in energy, environmental protection, economic, social development, and other fields can be carried out.

**Fig 2:**
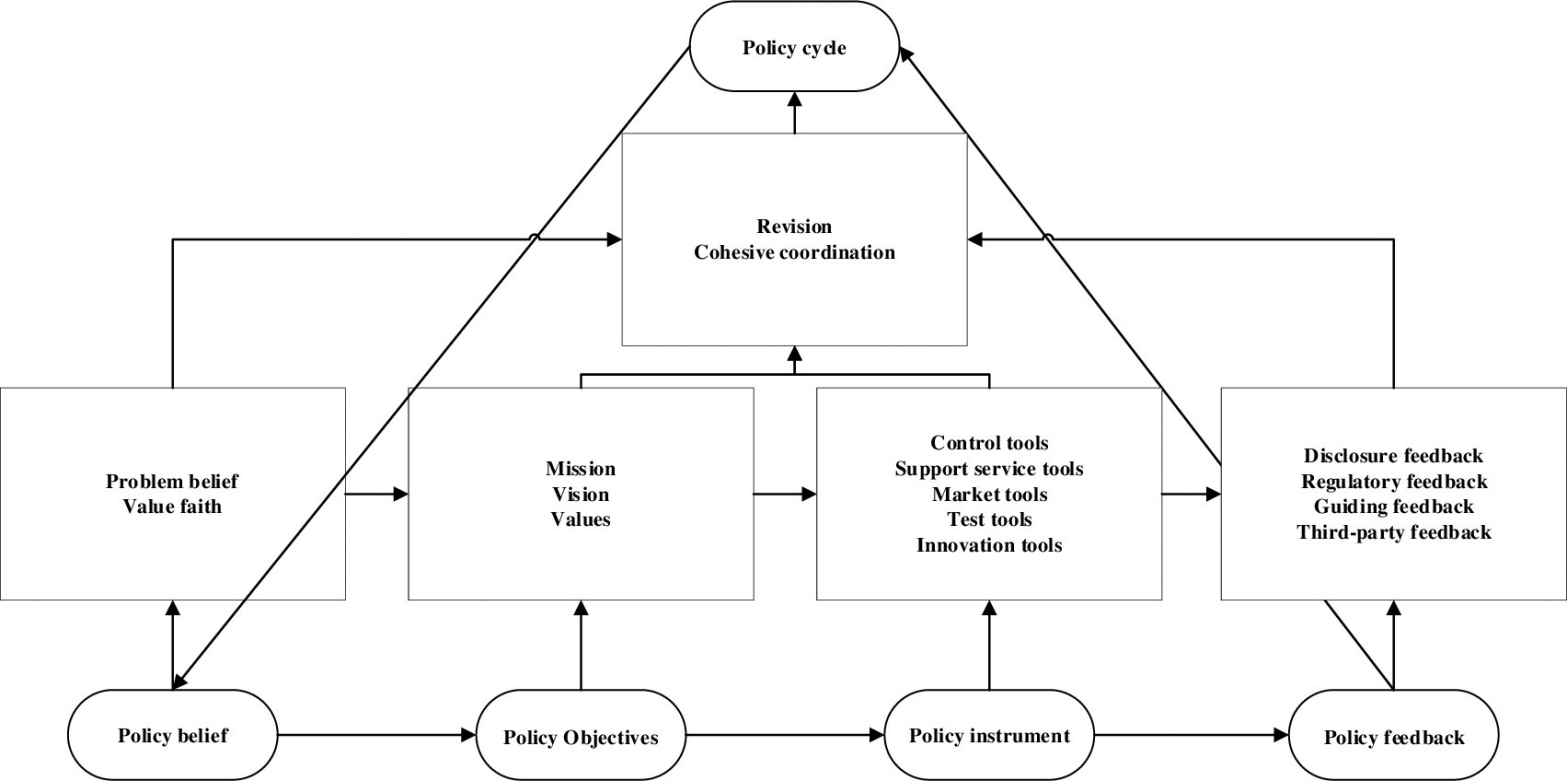
China’s green finance policy governance model.

Based on the above analysis, China’s green finance policy governance model can be constructed as shown below (see Fig 2).

### 4.2 Theoretical saturation test

In a theoretical saturation test, when no new attributes of a category derived from theoretical sampling data emerge, the attributes of the category are considered saturated and theoretical saturation has been reached [70]. We conducted a coding analysis of the remaining three policy texts, according to grounded theory procedure, in order to test for theoretical saturation. The results indicated that the categories overlapped and resembled each other, and that no new important categories and relations appeared. Thus, the categories in the model were quite abundant, and it can be considered that the constructed green finance policy governance process model was theoretically saturated.

For the content of the fourth part above, we refer to the content of relevant studies published by the authors [56].

### 4.3 Model interpretation

Through the governance logic model constructed according to the three steps above, it can be determined that policy beliefs derive policy objectives; policy objectives influence policy tools; the operational effect of policy tools influences policy feedback; policy beliefs, policy objectives, policy tools, and policy feedback all influence the policy cycle; and the governance practice of green finance policy presents a circular progression under the mutual influence of these categories, where incremental reforms must be carried out until the policy objectives are achieved. The respective constitutive dimensions of these five categories are detailed in the following.

#### 4.3.1 Policy belief

Policy belief is the logical starting point of China’s green finance policy governance process, playing a thought-leading role in the entire process of green finance policy governance under the influence of the external environmental situation. From the perspective of content composition, it is composed of two categories: problem belief and value belief. Problem belief refers to the conviction that the climate and environment situation facing the policy target poses an objective threat to human survival and development. This causes green finance to enter the vision of policy governance, and it is included in the scope of governmental functions. It is a necessary cognitive process driving policy activities and processes. Meanwhile, value belief is a policy object based on the scientific judgment of problem belief, where the policy process is based on affirming cognition and judgment, which makes the implementation of green finance policy meaningful.

In the process of sorting out China’s green finance policy texts, we observed that the policy subjects had realized the importance of constructing and improving the green finance governance system, as well as the urgency of serving both economic development and environmental protection economic activities in the context of the severe climate warming environment situation, then consequently adopted a series of publicity and education activities required by the policy. In this line, the consensus of developing green finance in our country was reached through the object of policy execution.

#### 4.3.2 Policy objectives

Policy objectives denote the mission, vision, and value underlying China’s green finance policy development. Driven by policy beliefs, they are a set of specific missions, visions, and values that can be measured and evaluated, either qualitatively or quantitatively. It is the action guide and main driver of subsequent policy links. From the perspective of content composition, three main aspects can be observed: mission, vision, and values. Mission refers to the responsibilities of the subject and object of policies in dealing with climate change and promoting green and high-quality development, which affects the direction of policies and directly determines the direction of policy vision.

In the process of sorting out China’s green finance policy, we observed that the current green finance policy is mainly focused on the concept of green development, supporting services for economic activities that have both economic and environmental effects, and promoting the core policy goal of high-quality green transformation, through the two policy visions of "carbon peak" and "carbon neutralization". The so-called carbon peak means that China promises to stop the growth of carbon dioxide emissions before 2030, then to slowly reduce them after reaching the peak. Carbon neutrality means that, by 2060, China has promised to offset all carbon dioxide emissions by planting trees, saving energy, and reducing emissions. The process of policy vision gradually becoming reality is the evolution process of the policy subject and object realizing policy goals according to mission and value judgments.

#### 4.3.3 Policy tools

Policy tools refer to a combination of policy means adopted by the government to achieve policy objectives, and they are the most critical link in the process of China’s green finance policy governance. This is because they comprise the process through which the government implements policy objectives with policy beliefs, and are also the links that need to be referred to in the following policy feedback. The real process involves testing the enforceability of policy texts, whether the policy implementation subjects are firm in their policy beliefs and, more importantly, determining the complexity of the interest demands of different policy subjects. It is the most active, uncertain, and complicated key link in the whole process of green finance policy governance in China.

In the process of sorting out China’s green finance policy, we observed that China has conducted a lot of exploration and made many attempts regarding green finance policy in recent years, and a certain degree of progress has been made at the level of policy text; however, its influence at the level of practical operation remains to be investigated. Green finance is considered the only means of simultaneous green transformation and sustainable financial development, posing a crucially important overall problem. The promotion of green finance depends on a good financial environment and infrastructure. The development imbalance between Chinese regions is prominent, and the financial environment and infrastructure vary widely, making it difficult to promote green finance. Compared to promotion, policy transmission path innovation is very important, and how to implement policy transmission path innovation and the development of green finance with local characteristics are key difficulties for green financial policy system construction in China.

#### 4.3.4 Policy feedback

Policy feedback is an important part of China’s green finance policy governance process, which is of great significance for improving policy quality and promoting policy coordination. It mainly consists of four categories: disclosure feedback, supervision feedback, guidance feedback, and third-party feedback. Policy feedback allows for the realization of policy objectives and improvement of the effectiveness of policy tools.

In the process of sorting out China’s green finance policies, we observed that the policy feedback process of China’s green finance policies is generally carried out by strengthening information disclosure and sharing, strengthening supervision and management, introducing incentive and restraint measures, and promoting third-party support. At present, there are many mandatory and rigid policy feedback measures, while soft incentive and reward policy feedback is insufficient, and should be strengthened in the future.

#### 4.3.5 Policy cycle

Policy cycle refers to the activities and processes by which the government adjusts, improves, and modifies policies according to the policy tools and policy feedback results, in order to more effectively achieve policy goals under the guidance of policy beliefs. It forms the inflection point for the closed-loop management of green finance policy within a certain policy time limit. Therefore, it is not only an end-point but is also the starting point; that is, it is a link that is always associated with policy beliefs, policy objectives, policy tools, policy feedback, and other policy links. It allows for a progressive revision and improvement process, facilitating a cyclic trend. It is a very important link, but is typically poorly governed and prone to the “policy circle” phenomenon: the so-called policy circle involves the recycling of policy text with no substantial improvement content, leading policy governance to become the use of policy text to govern policy text, and further leads to the illusion of policy belief, the loss of policy objectives, the contradiction and disorder of policy tools, and obstruction of the policy cycle. Overall, this forces the whole policy governance process into formalism. This should be paid attention to in China’s future green finance policy governance, in order to avoid this phenomenon as much as possible. At the same time, green finance policies involve various fields, such as the environment, energy, economy, and social development. Addressing the fragmentation of China’s green finance policies and how to achieve policy cohesion are key issues in this regard.

## 5. Clear set qualitative comparative analysis

As a qualitative comparative analysis method, clear set qualitative comparative analysis (csQCA) can be applied when considering 10–60 samples. Based on further verification and extension of the grounded theory research results, we took 19 policy texts with grounded coding as the research object to further study the influence of China’s green finance policy. The policy influence referred to in this paper denotes the force generated by a certain policy within the system; that is, if a certain green finance policy is used as the basis for other green finance policies, it is considered to have an influence within the green finance policy system.

### 5.1 The setting of explanatory variables

Based on the previous rooted coding analysis results, we took the five components of China’s green finance policy governance model as explanatory variables. First, with the help of the matrix coding function of the NVivo12plus software, a coding matrix was obtained, with the 19 policy text cases as rows and the 5 governance components as columns (see Table 4). Then, according to the bipartite attribution principle, the mean value method was used to assign values of "1" or "0" to the explanatory variables.

**Table 4:** Setting of explanatory variables for green finance policy.

No.	Policy Beliefs	Policy objectives	Policy tools	Policy feedback	Policy cycle
NA	1	5	10	15	1
NB	1	2	5	8	0
NC	0	3	3	22	0
ND	4	2	41	30	1
NE	1	3	34	13	0
NF	0	1	10	2	0
NG	0	1	23	7	0
NH	1	1	4	4	0
NI	1	1	67	15	0
NJ	2	2	23	11	0
NK	2	1	23	9	1
NL	0	3	44	2	0
NM	0	1	15	1	0
NN	1	1	7	1	2
NO	0	2	29	1	0
NP	0	0	5	2	0
NQ	0	2	20	10	0
NR	0	0	0	0	0
NS	0	0	0	0	0
Average	0.74	1.63	19.11	8.05	0.26

### 5.2 Setting of the result variable

Based on the above rooted coding analysis results, we took whether a green finance policy was the basis of other green finance policies as the standard; that is, those which serve as the basis for other green finance policies were assigned a value of 1; otherwise, they were assigned a value of 0. From the results, the policy texts numbered NC, ND, NG, NJ, and NQ were assigned a value of 1 (see Table 5 for the corresponding coding reference points), while the others were assigned a value of 0.

**Table 5:** Basis for setting the result variable.

No.	Policy name	Reference points
NC	Notice of the People’s Bank of China on Matters related to strengthening the supervision and administration of the duration of Green Finance Bonds	1
ND	A guideline on building a green finance system from the People’s Bank of China, the Ministry of Finance, the Development and Reform Commission, etc	3
NG	Announcement of the People’s Bank of China (2015) No. 39—Announcement on matters related to the issuance of green finance bonds in the interbank bond market	4
NJ	China Banking Regulatory Commission Circular on issuing green credit guidelines	2
NQ	Announcement of the China Interbank Market Dealers Association No. 10 [2017]—Announcement on the release of the Business Guidelines on Green Debt Financing Instruments for Non-Financial Enterprises and supporting forms	1

### 5.3 Truth table construction

After assigning values to the explanatory and result variables, we constructed a truth table, according to the QCA research steps, as shown in Table 6. For example, the truth table code of the explanatory variable in the case NA was 11011 (Table 6; from left to right). This means that, in case NA, the green finance policy mainly carries out green finance governance through policy belief, policy objective, policy feedback, and policy cycle governance logic. The truth table code for the final result variable was 0 for the NA case, which means that this green finance policy has no policy influence under the mixed application of the four abovementioned governance elements; that is, a policy containing the above four governance elements had not been used as a basis to influence the formulation of other green finance policies in the green finance policy system.

**Table 6:** Truth table for variable combinations of different green finance policy cases.

Case	Policy Beliefs (BE)	Policy objectives (AI)	Policy instrument (TO)	Policy feedback (FE)	Policy Cycle (CY)	Result Policy influence (IN)
NA	1	1	0	1	1	0
NB	1	1	0	0	0	0
NC	0	1	0	1	0	1
ND	1	1	1	1	1	1
NE	1	1	1	1	0	0
NF	0	0	0	0	0	0
NG	0	0	1	0	0	1
NH	1	0	0	0	0	0
NI	1	0	1	1	0	0
NJ	1	1	1	1	0	1
NK	1	0	1	1	1	0
NL	0	1	1	0	0	0
NM	0	0	0	0	0	0
NN	1	0	0	0	1	0
NO	0	1	1	0	0	0
NP	0	0	0	0	0	0
NQ	0	1	1	1	0	1
NR	0	0	0	0	0	0
NS	0	0	0	0	0	0

Data source: Collated and obtained by the authors according to the actual situation of 19 cases.

### 5.4 Research results and analysis

#### 5.4.1 Univariate necessity analysis

Qualitative comparative analysis is conducted to study cases with "many causes and one effect,” in which it is necessary to test whether each factor can independently influence the outcome. If each explanatory variable in the case can independently influence the outcome variable, a QCA method cannot be used. Therefore, we conducted a univariate necessity analysis for the condition variables in the selected cases.

After the truth table was constructed, "XY plot" was run in Tosmana software, in order to test whether there was a single explanatory variable that had an influence on the result variable; that is, in order to analyze whether there exists a sufficient or necessary relationship between each single explanatory variable and the result variable. Consistency and coverage are used to test for the above relationships, respectively, in the QCA method.

The judgment principle of consistency is as follows: If the consistency is greater than 0.8 and less than 0.9, the single conditional variable X is a sufficient condition for the result variable Y. If it is greater than 0.9, the single condition variable X is a necessary condition for the result variable Y, and vice versa. The coverage result is used to explain the explanatory power of the combination of condition variables on the result variable Y: the higher the coverage rate, the more ability X has to explain Y.

As shown in Table 7, the consistency values of the five condition variables were all below 0.8, indicating that these five variables did not independently affect the green finance policy; that is, the influence on green finance policy was the result of the combined action of various policy factors, rather than being determined by a single policy factor. Therefore, we continued our analysis, in order to determine an effective combination of variables, through which the influence on green finance policy can be enhanced.

**Table 7:** Univariate consistency and coverage.

Variable names	Consistency	Coverage
Policy Beliefs (BE)	0.2222	0.4000
Policy Objectives (AI)	0.4444	0.8000
Policy Tools (TO)	0.4444	0.8000
Policy Feedback (FE)	0.5000	0.8000
Policy Cycle (CY)	0.2500	0.2000

Data source: Tosmana calculation results

#### 5.4.2 Results of qualitative comparison and theoretical analysis

**Fig 3:**
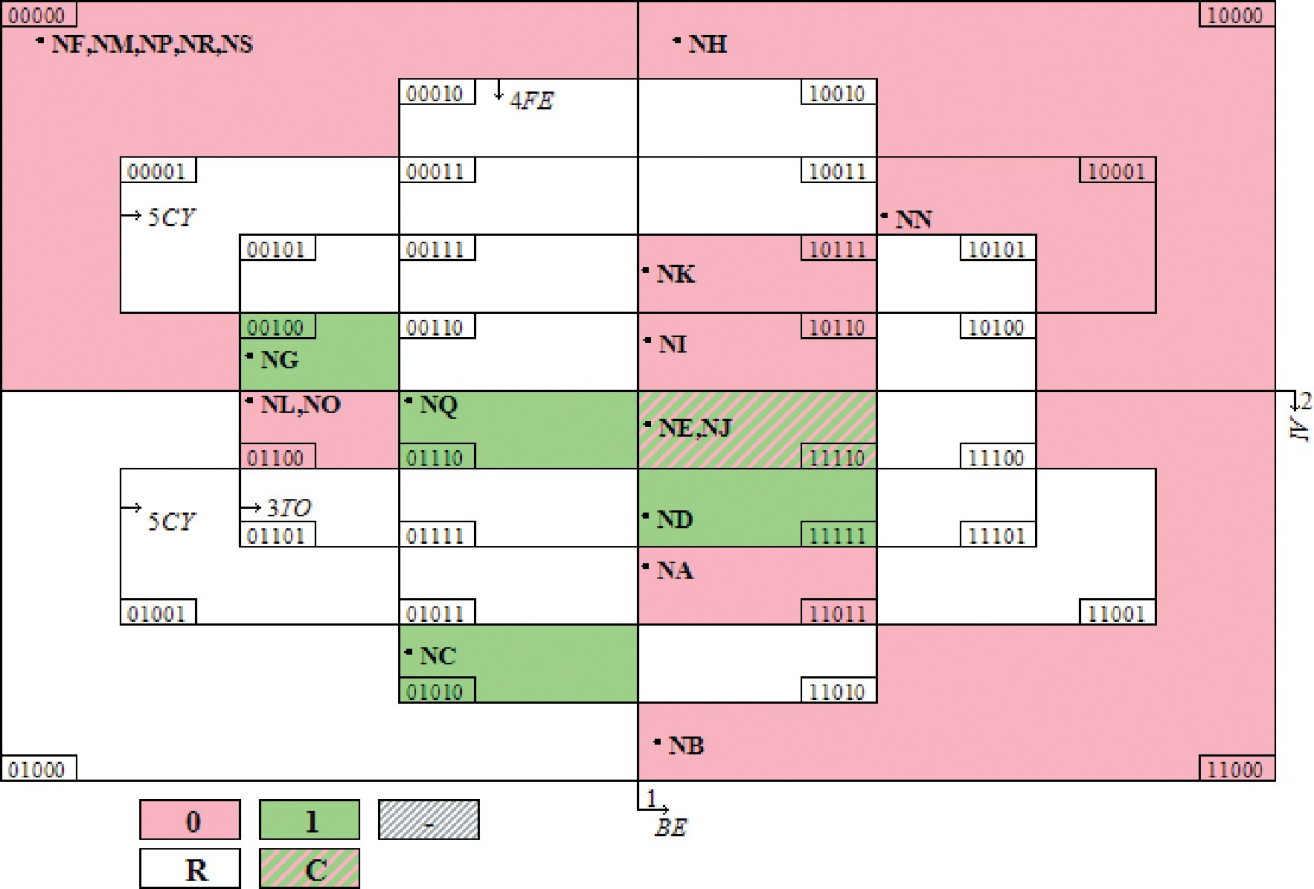
Results of qualitative comparative analysis.

After the necessity test was passed, the truth table (in Excel form) was saved in.csv format and the saved file was imported into Tosmana. Then, "Start csQCA" was run to obtain the combinations of policy elements influencing green finance policy. After calculation, the Tosmana software returned four configuration presentation modes; namely, Configuration 1, Configuration 0, Configuration C, and configuration R (as shown in Fig 3). In brief, Configuration 1 is the combination of policy elements that make a green finance policy influential; Configuration 0 refers to the combination of policy elements that cannot make a green finance policy influential; Configuration C is the contradictory configuration; and Configuration R is the logical remainder. The combined path of Configuration 1 and Configuration 0 is provided in Table 8.

**Table 8:** Analysis of qualitative comparison results of combination paths comprising different green finance policy elements.

Path combination	coverage	consistency
Path 1 be * AI * FE * cy	0.4000	1.0000
Path 2 BE * AI * TO * FE * CY	0.2000	1.0000
Path 3 be * ai * TO * fe * cy	0.2000	1.0000

The result of Configuration 1 is calculated as follows:

Result = be * AI * FE * cy+BE * AI * TO * FE * CY+be * ai * TO * fe * cy,

while the result of Configuration 0 is calculated as

Result = BE * to * fe * cy+ai * to * fe * cy+BE * ai * to * fe+BE * ai * TO * FE +BE * AI * to * FE * CY+be * AI * TO * fe * cy.

(The "*" in the publicity represents the "and" in the intersection, with uppercase for presence and lowercase for non-presence).

As can be seen from the qualitative comparison results in Table 8, Path 1 had the highest coverage rate, indicating that, compared with Paths 2 and 3, Path 1 is the policy element combination model that makes green finance policy more influential. The corresponding policy case is NC; namely, the Notice of the People’s Bank of China on Matters related to Strengthening the Supervision and Management of the Duration of Green Finance Bonds. Therefore, current green finance policies in China are still dominated by management-oriented policies, and the basic role of the market has not yet been given full play. Overall, the three paths obtained through the clear set qualitative comparative analysis are the main combination paths describing the influence of China’s green financial policy. The detailed analysis is as follows:

#### Path 1: Regulation-oriented

The resulting formula is (~ policy belief) * (policy objective) * (policy feedback) * (~ policy cycle); that is, be * AI * FE * cy (where "~" stands for non, equivalent to a lowercase letter combination).

Path 1 indicates that, even though the current green finance policy in China fails to convince the policy target of the importance and necessity of developing green finance, nor does it revise and improve the policy, it does clarify the specific mission, values, and vision of the policy in the form of text, as well as the specific assessment criteria and incentive and constraint mechanisms. This green finance policy also has a great influence within the system. The path shows that, on one hand, the mobilization of the policy object is not strong at present, and the enthusiasm and initiative of the policy object cannot be fully brought into play. On the other hand, the responsiveness of China’s green financial policy to practice is insufficient at present, such that the policy cannot be revised and improved in time, according to the exploration of green financial practice, which is in urgent need of strengthening in the future.

#### Path 2: Collaborative-driven

The resulting formula is (policy belief) * (policy objective) * (policy instrument) * (policy feedback) * (~ policy cycle); that is, BE * AI * TO * FE * CY.

Path 2 indicates that the influence of our current green finance policy can be brought into play under the condition that the policy belief is firmly established, the policy goal is clear, various policy tools are well-used, and policy feedback and policy cycle are effectively implemented. The target policy case is ND; namely, the Guiding Opinions of the People’s Bank of China, the Ministry of Finance and the National Development and Reform Commission on the Construction of green Finance System. This indicates that this policy—as a programmed policy of the green finance policy system—exerts an influence to a certain extent; however, the coverage rate of this path is not high, indicating that the influence of this policy needs to be improved. The path indicates that there are few policies that take into account the elements of green finance policy, and most green finance policies do not effectively take the five policy governance elements into account. Therefore, the synergistic effect of policy elements needs to be enhanced.

#### Path 3: Instrument-oriented

The resulting formula is (~ policy belief) * (~ policy objective) * (policy instrument) * (~ policy feedback) * (~ policy cycle); that is, be * ai * TO * fe * cy.

Path 3 indicates that the current green financial policy exerts some influence within the system of green financial policy, even when we only take the policy tools into account. This path indicates that policy tools are very important constituent elements of green financial policy in China. If a green financial policy cannot give consideration to other modules, it must make considerable efforts regarding policy tools. This result fully verifies that policy tools are the most critical links in the process of China’s green financial policy governance. The corresponding policy case of this path is NG; namely, the Announcement of the People’s Bank of China (2015) No. 39—Announcement on Issues Related to the issuance of Green Financial Bonds in the inter-bank Bond Market. The policy, issued by the People’s Bank of China in December of 2015, has milestone significance in China’s green financial market, as it provides guidance for the issuance of green financial bonds in the inter-bank bond market. As such, its release marked the official launch of China’s green bond market.

In Fig 3, pink represents configuration 0. Different from configuration 1, from which the path combinations that can exert an influence on green finance policy may be obtained, configuration 0 shows which policy element combination paths cannot exert an influence on green finance policy. Overall, we used clear set qualitative comparative analysis to determine six policies that hinder green finance from exerting its influence. The specific analysis is as follows:

~ Path 1: (policy beliefs)* (~ policy tools)* (~ policy feedback)* (~ policy cycle); that is, BE * to * fe * cy.

~ Path 1 indicates that, if a green finance policy is only established according to the policy object, without the organic coordination of policy tools, policy feedback, and policy cycle, the green finance policy cannot exert its influence well. This path indicates that policy belief is a soft element in the system of green finance policy, and is insufficient in supporting the policy to exert its due influence. The corresponding cases of this path are (NB+NH); namely, the Notice of the People’s Bank of China on Issuing the Green Finance Evaluation Plan for Banking Financial Institutions and the Notice of the China Interbank Market Dealers Association on Strengthening the Self-discipline Management of Green Finance Bond Duration Information Disclosure. These two green finance policies in China’s green finance policy system mainly play roles in cultivating the awareness of policy objects to develop green finance and guiding market institutions, in order to strengthen the self-discipline management of information disclosure, and have a limited influence on the whole green finance policy system.

~ Path 2: (~ policy target)* (~ policy instrument)* (~ policy feedback)* (~ policy cycle); that is, ai * to * fe * cy.

~ Path 2 indicates that, if a green finance policy is not clear in the links of policy objectives, policy tools, policy feedback, and policy cycle, it will be difficult for the green finance policy to exert influence in the green finance policy system. The cases corresponding to this path are (NF, NM, NP, NR, NS+NH); namely, the Notice of the People’s Bank of China on Supporting the issuance of Green Debt Financing Instruments in the Pilot Zone of Green Finance Reform and Innovation, the Guidance of the China Securities Regulatory Commission on Supporting the Development of Green Bonds, the Notice of the China Interbank Market Dealers Association on Matters related to Evaluating Certification Institutions’ Business of Green Debt Financing Instruments, and the Guidance on Financial Institutions’ Environmental Information Disclosure "Environmental Rights Financing Instruments" and "Notice of China Interbank Market Dealers Association on Strengthening the self-regulatory management of Green Finance Bond Duration Information Disclosure." These guiding and standard policies in the green finance policy system were designed to convey normative information, thus not placing them in a core position regarding the top-level design of green finance policy in China.

~ Path 3: (Policy belief)* (~ policy objective)* (~ policy instrument)* (~ policy feedback); that is, BE * ai * to * fe.

~ Path 3 indicates that, if a green finance policy only contains policy belief, but does not contain the policy objective, policy instruments, and policy feedback, its influence will be hindered. The cases corresponding to this path are (NH+NN); namely, the Notice of China Interbank Market Dealers Association on Strengthening the self-discipline management of Green Finance Bond Duration Information Disclosure and Notice of the People’s Bank of China, the Development and Reform Commission, and the China Securities Regulatory Commission on Issuing the Catalogue of Projects Supported by Green Bonds (2021 Edition), indicating the content composition of the above two green finance policies. They place emphasis on the establishment of policy beliefs on policy objects, and include no clear provisions regarding specific policy objectives, policy tools, and policy feedback, thus limiting their influence on the green finance policy system. Since the People’s Bank of China issued the Notice on Strengthening the Supervision and Management of Green Finance Bond Duration in 2022, the China Interbank Market Dealers Association has continuously monitored and evaluated the information disclosure of green financial bonds, and found that most issuers of green financial bonds disclose reports on the use of raised funds on time, in accordance with relevant regulations, which some issuers have presented non-standard disclosure content. In order to further improve the transparency of information disclosure of green financial bonds, complementary consolidation policies have been issued to help issuers to improve the quality of information disclosure and better serve the development of green finance. In this sense, such policy is designed to consolidate and supplement the upper policies, while occupying a core position in the central level of green finance system, thus having limited influence. The Notice on Issuing the Catalogue of Projects Supported by Green Bonds (2021 Edition) is a guiding policy formulated by the People’s Bank of China, the National Development and Reform Commission, and the China Securities Regulatory Commission, in order to improve the standardization and effectiveness of the support of green bonds for green projects, which is conducive to the implementation of green finance policies and the consolidation of policy beliefs. This policy also has limited influence on the top-level design of green finance in China.

~ Path 4: (Policy belief)* (~ policy objective)* (policy instrument)* (Policy feedback); that is, BE * ai * TO * FE.

~ Path 4 indicates that, even if a green finance policy contains three key components (i.e., policy belief, policy instrument, and policy feedback), the absence of policy objective will hinder its influence. The cases corresponding to this path are (NI+NK); namely, the Notice of the General Office of the China Banking Regulatory Commission on the Issuance of the Key Evaluation Index of the Implementation of Green Credit and the Opinions of the General Office of the China Banking Regulatory Commission on the Work of Green Credit. The above two green finance policies consolidate policy belief by regulating green credit—the main tool of green finance—and provide clear provisions for policy tools and policy feedback. However, they do not put forward clear policy objectives, such that their influence in the central level of green finance policy system has not yet been given full play.

~ Path 5: (Policy belief)* (policy objective)* (~ policy instrument)* (Policy feedback)* (Policy cycle); that is, BE * AI * to * FE * CY.

~ Path 5 indicates that, even if a green finance policy contains the four components of policy belief, policy objective, policy feedback, and policy cycle, it cannot have policy influence without the inclusion of policy tools. This path proves, from the opposite side, that policy tools are the most critical link in China’s green finance policy governance process, and is the most important among the five considered components. The corresponding case for this path is NA; that is, the Notice on Printing and Printing Green Finance Guidelines for the Banking and Insurance Industries. This policy is a guiding green finance policy, formulated by the China Banking and Insurance Regulatory Commission in June 2022 for all banks and insurance institutions. This policy contains less content, in terms of policy tools, resulting in its insufficient influence on top-level green finance design in China.

~ Path 6: (~ Policy belief)* (Policy objective)* (policy instrument)* (~ policy feedback)* (~ policy cycle); that is, be * AI * TO * fe * cy.

~ Path 6 indicates that, if a green finance policy only contains policy objectives and policy tools, it will have difficulty in exerting its influence without links to policy belief, policy feedback, and policy cycle. The cases corresponding to this path are (NL, NO); namely, the Announcement of the People’s Bank of China and the China Securities Regulatory Commission (2017) No. 20—Guidelines on Green Bond Evaluation and Certification (Interim) and the Notice of the General Office of the National Development and Reform Commission on Issuing Guidelines on Green Bond Issuance, Although the above two policies clearly define policy objectives and policy tools, the policy objects lack firm policy belief, and there is no perfect policy feedback mechanism. Therefore, it is impossible to assess the degree of the realization of policy objectives and the effectiveness of policy tools, such that closed-loop management cannot be realized. Furthermore, they do not give consideration to the revision and improvement of policies. As a result, the influence of the above policies in China’s green financial policy system is weak.

## 6. Conclusion

This paper mainly consisted of two parts: First, we used the NVivo12plus software and drew lessons from grounded theory to conduct rooted coding analysis of 22 existing green finance research policy texts in the Chinese context. Second, on the basis of the rooted coding results, the Tosmana software was used to carry out comparative analysis regarding the influence of central green finance policies, based on the clear set qualitative comparative analysis method.

The results of the rooted coding research demonstrated the following:

1. Policy belief is the logical starting point of China’s green finance policy governance process, which plays a thought-leading role in the overall process of green finance policy governance under the influence of the external environmental situation. Policy belief consists of problem belief and value belief.
2. Policy objectives denote the mission, vision, and values inherent to China’s green finance policy development. Driven by policy beliefs, it is a specific set of missions, visions, and values that can be measured and evaluated, either qualitatively or quantitatively, and guides the action of subsequent policy links. Here, mission refers to the responsibilities and responsibilities of the subjects and objects of policies in addressing climate change and promoting green and high-quality development. policy objectives affect the direction of policies and directly determine the direction of policy vision.
3. Policy tools denote the combination of policy means adopted by the government to achieve policy objectives. They are the most critical link in the process of China’s green finance policy governance, as they comprise the means by which the government will implement policy objectives driven by policy belief, while also serving as the key reference in the following policy links. The actual process involves testing the implementation ability of the policy text, whether the policy implementors are firm in their policy beliefs or not and, more importantly, determining the complexity of the interest demands of different policy subjects. This is the most active, uncertain, and complicated key link in the whole process of green finance policy governance in China.
4. Policy feedback is an important part of China’s green finance policy governance process, which has great significance for the improvement of policy quality and the promotion of coordinated policy operation. It mainly consists of four sub-categories—disclosure feedback, regulatory feedback, guidance feedback, and third-party feedback—and allows for the realization of policy objectives and improvement of the effect of policy tools.
5. Policy cycle is an activity and process in which the subject and object of policy adjust, improve, perfect, revise, and connect policies, according to the policy tools and policy feedback results, in order to achieve policy goals under the guidance of policy beliefs. It forms the inflection point of the closed-loop management of green finance policy within a certain policy time limit. Therefore, it is not only the end-point, but also the starting point, and may be considered as a link associated with policy belief, policy goal, policy tool, and policy feedback categories at any time. It involves a process of gradual revision and improvement, presenting a cyclic trend. It is a very important link, but is also one which is typically poorly governed and prone to the policy circle phenomenon (i.e., recycling policy text with no substantive improvement content), which causes policy governance to devolve to the use of policy text for the governance of policy text, and may even lead to the absence of policy belief, the loss of policy objectives, the contradiction and disorder of policy tools, and obstruction of the policy cycle. Overall, the whole process of policy governance becomes formalism, which should be avoided as much as possible. Green finance policy involves various other fields, including the environment, energy, economics, and social development. Addressing the fragmentation of China’s green finance policy and how to achieve policy cohesion is a crucial issue.

The clear set qualitative comparative analysis results show the following:

1. There exist three combination paths with an influence on green finance policy: Path 1: (~ policy belief) * (policy objective) * (policy feedback) * (~ policy cycle); Path 2: (~ policy belief) * (policy objective) * (policy instrument) * (policy feedback) * (~ policy cycle); and Path 3: (~ policy belief) * (~ policy objective) * (policy instrument) * (~ policy feedback) * (~ policy cycle). Among them, Path 1—namely, the combination pattern of non-policy beliefs, policy objectives, policy feedback, and non-policy cycle—is the combination pattern of policy elements that is the most influential in China’s green finance policy.
2. Policy tools are the most critical link in the governance process of China’s green finance policy.
3. At present, China’s green finance policies are still dominated by management-oriented policies, and the basic role of the market has not yet been given full play.
4. To date, the mobilization of the target of the green financial policy in China has not been strong, and the enthusiasm and initiative of the targets of the policy cannot be fully brought into play.
5. The responsiveness of China’s current green financial policy to practice is insufficient, such that it cannot be revised and improved in a timely manner, according to the required exploration of green financial practice. This aspect is in urgent need of strengthening in the future.
6. At present, there are few policies that give consideration to the key components of green finance policies, as most green finance policies do not give consideration to the five policy governance elements considered here. Therefore, the synergistic effects of policy elements need to be enhanced.

## 7. Countermeasures and prospects

Through grounded theory tracing and clear set qualitative comparative analysis, we derived the following policy suggestions, which may promote the development and improvement of China’s green finance policy.

### 7.1 Innovate policy mix and improve policy stimulus

Green development relies on green projects, which have high cost and high risk, and are not conducive to mobilizing the initiative and enthusiasm of financial institutions in developing green finance. Therefore, the government needs to innovate the policy mix, guide and supervise in a timely manner, and focus on improving the stimulus effect of policies. On one hand, it is necessary to strengthen top-level design, scientific accounting, and introduce incentives that are actually attractive to financial institutions. A blueprint should be drawn to this end, such that financial institutions can truly enjoy the benefits brought by the development of green finance. On the other hand, all kinds of financial institutions should be encouraged to innovate the green finance management mechanism and introduce characteristic incentive and constraint policies, according to their own actual conditions. On the basis of not violating the overall national design, green finance policies should be improved to fit and respond to varying green finance practices, and the green finance policies should be continuously optimized and improved in a bottom-up manner.

### 7.2 Give full play to the main role of the market and improve the driving force of policies

In essence, the development of green finance is driven by the green finance market. From the perspective of public policy, it is necessary to break the fragmented governance pattern of green finance market, strengthen the integration of different policies (e.g., mandatory and supply), and build a good green finance market. On one hand, the definition and development of green projects should be promoted and standardized, in order to keep up with the development pace of endless new green products, as well as safeguarding the security and stability of the green finance market. On the other hand, the use and fund management of green funds should be regulated, increased disclosure of green finance information should be encouraged, and the market governance dilemma of green finance should be addressed, such that green finance can become a powerful engine which guides funds to the green market.

### 7.3 Appropriate legislation, reform, abolition, and improved promotion of policies

The development of green finance requires policies to match both spatially and temporally. In terms of the time dimension, green finance is currently in a stage of rapid development. On the basis of maintaining the stability of guiding policies, it will be necessary to improve the activities supporting green finance policies, revise and improve policies with strong implementation and evaluation based on green finance practices in a timely manner, and promote green finance policies to keep up with the times and to bring novel developments to the forefront. In terms of the spatial dimension, with the gradual progress of green finance pilot work, the pilot experience can be expected to gradually enrich the promotion of policy. Policy affecting various localities and subjects should be combined with their reality, avoiding blindly following suit and copying. Adaptive governance thinking should be established for the construction of green finance markets at the local level. Finally, experience should be continuously summarized in order to facilitate innovative policy design, thus improving upon the policy promotion power when considering homogeneous subjects.
